# Current News

**Published:** 1891-06

**Authors:** 


### Current News.

### COLORADO STATE DENTAL ASSOCIATION.

THE next annual session of the Colorado State Dental Association will be held in Denver, June 5–7, 1891.

J. H. PARSONS, D.D.S.,
*Corresponding Secretary.*

BOULDER, COL., April 9, 1891.

---

### MISSOURI DENTAL COLLEGE ALUMNI.

THE annual meeting of the Alumni Association of the Missouri Dental College was held March 12, 1891, at the College building, corner Seventh Street and Clark Avenue. Ten new members were elected to membership, including Drs. Newby and Wick, of this city, the others being members of the class of 1891.

Officers for 1891 were elected as follows: President, Dr. W. M. Bartlett; Vice-President, Dr. J. H. Prothero; Secretary, Dr. De Courcy Lindsley; Treasurer, Dr. Carl E. Schumacher; Executive Committee, Drs. Bowman, Prosser, and Whipple.

During the past year the following Alumni have been lost by death: Dr. Homer Judd, M. D. La Croix, John W. Forden, and Henry D. Field. The following committee were appointed to draft suitable resolutions regarding the foregoing, to be presented at the next annual meeting: Dr. W. H. Eames, A. H. Fuller, and John G. Harper. The by-laws were changed, doing away with annual dues.

---

### MISSOURI STATE DENTAL ASSOCIATION.

ST. LOUIS, MISSOURI, March 31, 1891.

THE Twenty-seventh Annual Meeting of the Missouri State Dental Association will be held at Louisiana, Mo., July 7, 8, 9, and 10, 1891.

Two half-days will be devoted to clinics,—Wednesday and Thursday. Those having new specimens, models, new appliances and methods, should notify the committee in order that proper mention of them can be made on the programme.

WM. CONRAD,<br>

J. W. WHIPPLE,<br>

HENRY FISHER,<br>

*Executive Committee.*

---

### CONNECTICUT VALLEY DENTAL SOCIETY.

The Twenty-seventh Annual Meeting of the Connecticut Valley Dental Society will be held at Hotel Hamilton, Holyoke, Mass., on June 10, 11, and 12. Arrangements already made indicate an interesting and profitable meeting. The clinics will be under the charge of Dr. L. D. Shepard, of Boston.

GEORGE A. MAXFIELD, D.D.S.,<br>

*Secretary.*

HOLYOKE, MASSACHUSETTS.

